# Body piercing and tattoos: a survey on young adults' knowledge of the risks and practices in body art

**DOI:** 10.1186/1471-2458-11-774

**Published:** 2011-10-07

**Authors:** Alessia Quaranta, Christian Napoli, Fabrizio Fasano, Claudio Montagna, Giuseppina Caggiano, Maria Teresa Montagna

**Affiliations:** 1 Department of Biomedical Science and Human Oncology - Hygiene Section - University of Bari "Aldo Moro", Piazza Giulio Cesare 11, 70124 Bari, Italy

## Abstract

**Background:**

The practice of tattooing and piercing has expanded in western society. In order to verify young adults' knowledge of the risk and practices related to body art, an investigation was conducted among freshmen of the University of Bari in the region of Apulia, Italy.

**Methods:**

The study was carried out in the Academic Year 2009-2010 through an anonymous self-administered written questionnaire distributed to 1.656 freshmen enrolled in 17 Degree Courses.

**Results:**

Of the 1.598 students included in the analysis, 78.3% believe it is risky to undergo piercing/tattoo practices. AIDS was indicated as a possible infection by 60.3% of freshmen, hepatitis C by 38.2%, tetanus by 34.3% and hepatitis B by 33.7% of the sample. 28.1% of freshmen were not aware that there are also non-infectious complications. 29% of the sample had at least one piercing or tattoo (this percentage does not include earlobe piercing in women). Of those with body art, the decision to undergo body art was made autonomously in 57.9% of the participants. 56.3% of freshmen undergoing body art had taken less than a month to decide. With regard to the reasons that led the sample to undergo body art, 28.4% were unable to explain it, 23.8% answered to improve their aesthetic aspect, 18.4% to distinguish themselves from others, 12.3% for fashion; 17.1% for other reasons. 25.4% of the sample declared that they had a piercing (79.8% female vs 20.2% male; ratio M/F 1:4.0). The average age for a first piercing was 15.3 years (range 10-27; SD ± 2.9). 9.6% of the sample declared that they have a tattoo (69.9% female vs 30.1% male; ratio M/F 1:2.3). The average age for a first tattoo was 17.5 years (range 10-26, SD ± 2.4).

**Conclusions:**

Most of the freshmen knew about AIDS-related risks but not other potential risks. Body art is fairly common among young adults (especially women). The decision is often not shared with the family and is undertaken mostly without a specific reason or for the improvement of aesthetic aspect. Information about freshmen's knowledge, attitudes and practices could help in effective planning of health promotion strategies.

## Background

The practice of tattooing and piercing, once not very common in developed countries, has now expanded into western society. In fact, in the last twenty years, young people have shown great enthusiasm for the practice [1-4]. Unfortunately, with this higher demand, the number of unprofessional tattooists and piercers has increased creating more complications due to frequent procedures carried out without any knowledge of health and hygiene rules [4,5]. The scientific literature shows a wide range of health consequences both infectious (e.g. HIV, HBV and HCV, mycobacterial infections, septicemia, abscess, endocarditis, tetanus) and non-infectious diseases (e.g. dermatitis, hemorrhage, allergies, damage to the oral cavity) [6-19]. There have been some cases with a fatal outcome [20]. A survey carried out in Great Britain has shown that 10% of the 10.503 subjects interviewed have piercing on another part of the body than the earlobes (nose, tongue, eyebrows, nipples and navel); the main complications have been oedemas, infections, hemorrhages and, in some cases, hospital treatments. Almost 10% of the subjects with a piercing on the tongue were performed by "non-specialists."; as a matter of fact in that paper, the tongue has turned out to be the part of the body where the majority of injuries related to piercing occurred [2]. Tattoos are also responsible for infectious complications: bacterial endocarditis [20], atypical mycobacterial infections [21], erythematic nodules [22]. In Italy, in April 1998, an appropriate intervention was promoted by the Ministry of Health to fight this phenomenon and the risks related through the publication of "Guidelines for the procedures of tattoos and piercing in health and safety conditions", whose last update was in 2004 [23].

The aim of the present study is to ascertain young Italian adults' knowledge, attitudes and practices with regard to the risk related to "tattooing-piercing" through an investigation among university freshmen in Bari, Southern Italy.

## Methods

### Study procedures

The study was carried out in the first half of the Academic Year 2009-2010.

Through a non-probabilistic quota sampling, 1.656 freshmen were selected among 17 Degree Courses of the University of Bari, Italy. The courses were grouped in three main fields: sanitary (Faculty of Medicine and Surgery), scientific (Faculty of Architecture, Engineering, Bio-Technological Science, Mathematics-Physics and Natural Sciences) and humanistic (Faculty of Literature and Science of Education). The selection of subjects to be enrolled in the study was performed carrying out the sampling until the fulfilling of each quota (at least 33%) for the three recruitment categories. The sample was asked to complete an anonymous questionnaire. All participants took part on a voluntary basis and were not remunerated for participation.

### The questionnaire

The form reporting the questions was divided into three sections:

1. Questions about age, sex, place of residence and parents' occupation. According to the "US Census Bureau" [24] occupations were classified in skill levels (skilled, semi-skilled, low/unskilled, unemployed).

2. Closed-ended questions about the participant's knowledge of the actual health risks linked to the practices of tattooing and piercing. Some questions allowed "yes"/"no"/"don't know" answers (e.g. "Is it risky undergoing piercing/tattooing?"; "Are the places and instruments used for body art always safe in terms of health and hygiene?"); other questions included the possibility of multiple choices (e.g. "If tattoos and piercing can transmit infectious diseases, which of those listed below?"; "Which of the non-infectious diseases listed below can follow a piercing and tattoo?").

3. Limited to people who underwent body art and distinct in two subsections: one for piercing and one for tattooing. Closed-ended questions with "yes"/"no"/"don't know" regarding advice required before practices, information given to the parents, giving informed written consent, acquisition of information regarding health risks. Closed-ended questions with multiple choices regarding: decision-making (including input from others), reasons for having the body art, place where the procedure was carried out; location of the tattoo/piercing, any complications reported. Open questions regarding the age at which the tattoo/piercing was carried out for the first time, and on how many piercings/tattoos young adults have.

### Data analysis

The data collected have been inserted in a database. Statistical analysis was executed by the Statistical Programme R version 2.8.0. Student's t-test was used to compare unpaired data; the χ2 statistical test was used to evaluate the association between independent variables. The relative confidence intervals at 95% were calculated and a value of p < 0.05 was considered significant for all the tests.

According to Schorzman et al [15], since in western society it is customary for women to wear earrings on both earlobes it was specified on the survey that, for women, piercing was to be considered "a metal object inserted in the skin in any part of the body, with the exception of the earlobes"; for men, instead, the ornament of the earlobes could be considered a piercing. The research does not report any experiment on human or biological human samples; it is an observational survey conducted by an anonymous questionnaire among freshmen approved by the *Apulia Regional Epidemiological Center *(Scientific Body of the Regional Health Authority).

## Results

Of the 1.656 enrolled freshmen, 1.598 returned a correctly filled out questionnaire (96.5%) and were considered for the analysis: 33.8% came from humanistic, 33.1% from healthcare and 33.1% from the scientific faculties.

Of the 1.598, 508 (31.8%) were male and 1.090 (68.2%) were female. The average age of participants was 20.15 years (range 17-58; SD = ±3.4); considering that in Italy the usual university freshmen age group ranges between 17-19 years, 62.7% (1.002/1.598) fell into this range. Of the students included in the analysis, 332 (20.8%) declared that they live in one of Apulia's five main towns (Bari, Brindisi, Lecce, Taranto, Foggia), while 1.266 (79.2%) lived in smaller municipalities. With regard to the profession of the father, the sample showed the following distribution: 60 (3.8%) unemployed, 442 (27.7%) low/unskilled worker, 667 (41.7%) semi-skilled, 429 (26.8%) skilled. With regard to the mother's occupations, the sample showed the following distribution: 112 (7.0%) unemployed, 870 (54.4%) low/unskilled worker, 513 (32.1%) semi-skilled, 103 (6.4%) skilled.

Of the 1.598 included in the analysis, 78.3% believe it is risky to undergo piercing/tattoo practices, 12.3% consider it not risky to undergo these practices and 9.4% don't know if it is risky or not (Table 1).

**Table 1:** Answers to questions about young adults' knowledge on health risks

Questions	Yes N° (%)	No N° (%)	Do not Know N° (%)	Tot N°
Is it risky undergoing piercing/tattooing?	1.251 (78.3%)	197 (12.3%)	150 (9.4%)	1.598

Can tattoos and piercing transmit infectious disease?	1.440 (90.1%)	48 (3.0%)	110 (6.9%)	1.598

Can tattoos and piercing transmit non-infectious disease?	1.040 (65.1%)	109 (6.8%)	449 (28.1%)	1.598

Are the places and instruments used for body art always safe in terms of health and hygiene?	114 (7.1%)	1.315 (82.3%)	169 (10.6%)	1.598

Is it possible to remove the tattoo?	1.395 (87.3%)	107 (6.7%)	96 (6.0%)	1.598

Is the piercing a permanent practice?	1.416 (88.6%)	62 (3.9%)	120 (7.5%)	1.598

In particular, with regard to infectious diseases, AIDS is indicated as possible infection by 60.3% of the whole sample included in the analysis (52.6%, 58.4% and 69.9% of the freshmen coming from humanistic, scientific and healthcare faculties respectively), hepatitis C by 38.2% (27.4%, 39.5% and 47.8% from humanistic, scientific and healthcare faculties respectively), tetanus by 34.3% (32.0%, 31.8% and 39.1% from humanistic, scientific and healthcare faculties respectively) and hepatitis B by 33.7% (23.5%, 32.7%, 45.0% from humanistic, scientific and healthcare faculties respectively).

Significant differences were showed in the data distribution when comparing freshmen from healthcare faculties vs those from the other two sectors: AIDS (χ2 = 30.4; p < 0.001), hepatitis C (χ2 = 30.61; p < 0.001), hepatitis B (χ2 = 72.75; p < 0.001), tetanus (χ2 = 7.90 p < 0.01).

Furthermore, 28.1% of the 1.598 freshmen were not aware that there are also non-infectious complications (allergies, scars, bleeding, etc.).

Of the 1.598 sample, 1.416 (88.6%) stated that the piercing is not a permanent practice and among those 92.1% think that the elimination of the piercing from the site of insertion leads to spontaneous closure of the insertion.

Of the 1.598 sample, 1.395 (87.3%) stated that it is possible to remove the tattoo, among those 59.9% by surgery (including laser surgery), 34.8% by subcutaneous aspiration of the ink, 5.2% by subcutaneous washing.

Of the 1.598 freshmen, 463 (29%) have at least one piercing or tattoo. Of those, 101 were male (21.8%) and 362 were female (78.2%) with a proportion M/F of 1:3.6. The difference between male (101 with body art/508 total male) and female (362 with body art/1090 total female) was statistically significant. (χ2 = 29.27; p < 0.001).

Of the 463 young adults who underwent body art, 96 freshmen (20.7%) confirmed that they have both piercing and tattoos.

Young adults belonging to humanistic faculties are more inclined than those belonging to healthcare and scientific faculties to undergo body art (χ2 = 19.67; p < 0.001); in particular 195/540 (36.1%) freshmen from humanistic faculties underwent body art vs 136/529 (25.7%) freshmen from the healthcare faculties and 132/529 (25%) from the scientific faculties.

74% of freshmen having body art were informed about the risks related to such practices before doing it (Table 2). The information came from the body artist (52%), another person (29.3%), reading the informed consent (18.7%).

**Table 2:** Answers to questions about young adults' attitudes and practices towards body art

Questions	Yes N° (%)	No N° (%)	Do not Know N° (%)	Tot N°
Have you been pierced?	406 (25.4%)	1.192 (74.6%)	0	1.598

If you don't have a piercing, would you consider it in the future?	249 (20.9%)	779 (65.3%)	164 (13.7%)	1.192

Have you been tattooed?	153 (9.6%)	1.445 (90.4%)	0	1.598

If you don't have a tattoo, would you consider it in the future?	525 (36.3%)	651 (45.1%)	269 (18.6%)	1.445

When you decided to undergo body art, did you ask someone's advise?	195 (42.1%)	268 (57.9%)	0	463

Were your parents informed when you underwent body art?	338 (73.1%)	125 (26.9%)	0	463

Did you sign any informed consent?	89 (19.3%)	368 (79.4%)	6 (1.3%)	463

Were you informed about the risks related to such practices?	343 (74.0%)	104 (22.6%)	16 (3.4%)	463

Did the operator use sterile/disposable instruments?	411 (88.7%)	12 (2.7%)	40 (8.6%)	463

Did you report any complication after the intervention?	61 (13.2%)	402 (86.8%)	0	463

**Figure 1:**
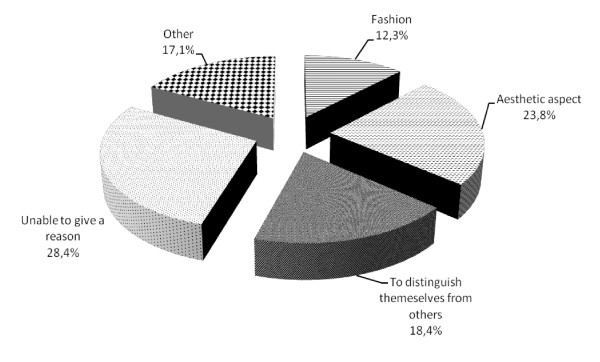
**Reasons for making a body art**. Others include: to emulate a familiar (3.1%); to better integrate in the society (4.5%); to feel better (6.5%), to better health conditions (0.6%); to follow a very important person (2.4%)

The decision to undergo body art was taken autonomously in 57.9% and asking the advice of someone in 42.1%. 56.3% of freshmen undergoing body art took less than a month to decide, 22.5% one to six months, 21.2% more than six months. With regard to the reasons that led the sample to undergo body art: to improve their aesthetic aspect (23.8%), to distinguish themselves from others (18.4%), for fashion (12.3%); 17.1% for other reasons; 28.4% of the interviewed was unable to give a reason (Figure 1).

**Figure 2:**
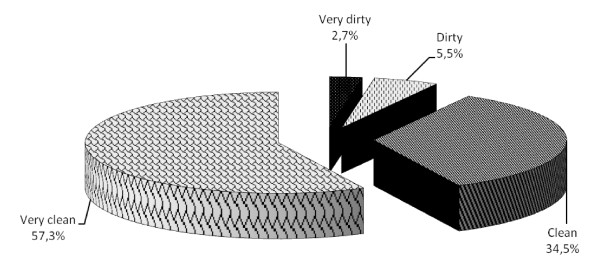
**Level of cleanliness of the place where the body art was carried out**.

With regard to the site carrying out the body art, 71.9% claimed they presented themselves to an authorized centre, 13% to a beautician, 7.1% to the cheapest place, 4.2% to a walking (street) artist, 3.9% declared they had performed the body art by themselves at home or at someone's house. In addition, 88.7% of the 463 who underwent body art stated that the instruments used were sterile and/or disposable and that the place was very clean (57.3%) (Figure 2).

**Figure 3:**
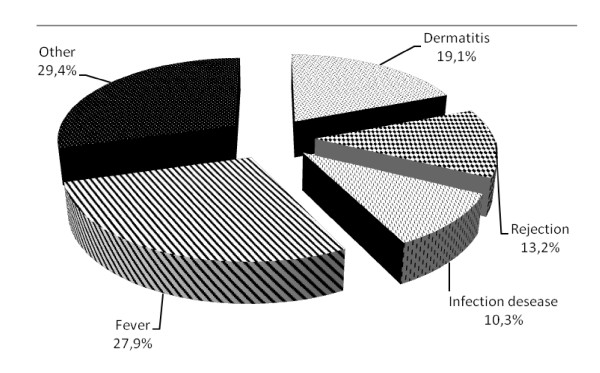
**Type of complications following the practices of piercing and tattoos**.

Sixty-one (13.2%) of the interviewees who underwent body art had had complications after it (Figure 3). Of the 61 who had experienced complications, 8 (13.1%) declared that they had had several symptoms at the same time. Furthermore, 9.2% of those who chose an authorized centre also had complications.

### Piercing

Of the 1.598 included in the analysis, 406 subjects (25.4%) declared that they have a piercing. Of the 406 pierced people, 324 were female (79.8%) and 82 were male (20.2%), with a ratio M/F 1:4.0. The difference between male and female was statistically significant (χ2 = 33.02; p < 0.001). A written informed consent, before the piercing, was required in 68/406 (16.8%).

The mean age at the first piercing was 15.3 years (range 10-27; SD ± 2.9). Of the 406 pierced freshmen, 314 (77.3%) did the piercing when they were underage (<18 years), and of those 214 (68.2%) informed their parents before the practice.

The mean number of piercings per pierced participant was 2.1 (range 1-16; SD ± 2.0). In particular, 54.6% of the 406 pierced freshmen confirmed they had only one, 24% two, 11.7% more than three, 9.6% three. There were no significant sex differences in the average number of piercings per person (p = 0.79). In addition, 84.3% decided to place the piercing on the head (including face, scalp and neck), 9.4% on the trunk and 6.3% on the limbs.

Among the interviewed who have stated that they have never had a piercing (74.6%), to the question "Would you consider it in the future?" 20.9% answered "yes", 13.7% "don't know", and 65.3% "no". A significant difference resulted between males and females: females showed a higher interest than males in a future piercing (χ2 = 16.10, p < 0.001).

Considering the variable of residence, of the 332 coming from main towns 168 (50.6%) had at least one piercing, while of the 1266 people coming from smaller municipalities 238 (18.8%) had at least one piercing. Those who live in one of Apulia's five main towns are more inclined than those who live in smaller municipalities to undergo piercing (χ2 = 140.37; p < 0.001).

The occupation of both mother and father does not affect the practice of piercing (father χ2 = 4.83; p = 0.18 - mother χ2 = 0.19; p = 0.98).

### Tattooing

Of the 1.598 sample, 153 freshmen (9.6%) declared that they have a tattoo. Of these 107 were female (69.9%) and 46 were male (30.1%), with a ratio M/F of 1:2.3. The difference between male and female was not statistically significant (χ2 = 0.10; p > 0.05). A written informed consent, before the tattoo, was required in 48/153 (31.3%).

The average age for the first tattoo was 17.5 years (range 10-26, SD ± 2.4). Of the 153 tattooed freshmen, 61 (39.9%) had the tattooing when they were still underage, and of those 39 (63.9%) informed their parents before the practice. The average number of tattoos per tattooed participant was 1.8 (range 1-17, SD ± 1.92). In particular, among the 153 freshmen admitted having a tattoo, 61.2% had only one, 23% two, 9.2% three, 6.7% more than three. There were no significant sex differences in the average number of tattoos (p = 0.11). In addition, 48.9% decided to place the tattoo on the limbs, 35.2% on the trunk and 15.9% on the head.

Among those who stated that they had never had a tattoo (90.4%), to the question "Would you consider it in the future?", 36.3% answered "yes", 18.6% "I do not know", and 45.1% "no". There was no significant difference between males and females with respect to interest in acquiring tattoos in the future: females did not show a higher interest than males in the future tattooing practice (male 156/306; female 369/614; χ2 = 1.93 - p > 0.05).

Considering the variable of residence, of the 332 coming from main towns 70 (21.7%) had at least one tattoo, while of the 1.266 people coming from smaller municipalities 83 (6.6%) had at least one tattoo. Those who live in one of Apulia's five main towns are more inclined than those who live in smaller municipalities to undergo tattooing (χ2 = 64.12 - p < 0.001). The occupation of both mother and father does not affect the practice of tattoos (father χ2 = 0.55; p = 0.91 - mother χ2 = 3.85; p = 0.28).

Student's t-test showed that average age for the first tattoo was significantly higher than the average age for the first piercing, even if the Cohen's d test indicated a large effect size (t = 8.4, p < 0.001; Cohen's d = 0.93).

## Discussion

The results of this study show that many young adults think it is risky to undergo piercing/tattoo practices. Interestingly, freshmen of healthcare faculties seem more careful about the issues of infectious complications related to body art practices. It might seem obvious that students of sanitary faculties know more of these aspects, if we did not consider the fact that this survey was conducted among freshmen, who might be supposed to have approximately the same level of knowledge. Nevertheless, this figure could be related to the fact that, in Italy, access to courses in a Faculty of Medicine requires a prior admission test on scientific matters. At the same time, young adults belonging to humanistic faculties seems more inclined than those belonging to the other two areas to undergo body art.

Regarding young adults' knowledge of infectious risks, the majority indicated AIDS as a possible infection risk, but not many people were aware of the risks associated with hepatitis B, tetanus and hepatitis C. This suggests that while AIDS-related risks are better known, other risks equally important need to be better specified and highlighted by health professionals, through information campaigns. Because there are significant health risks associated with piercing and tattooing, it is important to improve young adults awareness of them.

Although 90.1% of the sample was aware of the possible transmission of infectious diseases, 28.1% did not know that there are other kinds of risks, such as allergies, scars or bleeding. This figure could be considered an emerging problem of Public Health, in particular because the decision to undergo these procedures is often not shared with the family or experts in the field. As a matter of fact, encouraging young adults to talk with others (especially health professionals) about body art, asking specific questions and knowing enough is helpful to better judge the quality and hygiene of the artist activities [25]; thus, reducing the health risks.

Furthermore, for the most, the decision to undergo body altering is taken quickly (less than a month to decide) and most of the interviewed who underwent body art were unable to explain a reason for this choice. Both these findings are in agreement with those found in a survey conducted by Greif et al [25] in 1999 among 887 American college students.

The fact that, even if most of the whole sample stated that it is possible to remove a tattoo, only 59.9% knew that a surgical procedure is necessary to remove it, shows that young adults are not so well informed, as they perhaps believe. It has to be noted that the lack of a specific response about laser among those provided might have been confusing.

Many freshmen having body art claimed to have been informed about the risks related to such practices before entering them; the information came especially from the body artist.

According to Millner and Eichold [26], common themes for modifying bodies in the West include image management, sexual expression/sexual enhancement. In our study, the main reason given by young adults who underwent body art was related to the improvement of aesthetic aspect; smaller percentages are related to the need to be different from the others and to fashion. These motivations are in agreement with those having emerged from other investigations in this field [26-29].

The fact that 29% of the 1.598 sample have at least one piercing or tattoo and that 20.7% of those who underwent body art have declared they have both piercing and tattoos shows that body art is fairly common among young adults, as previous work confirms [26,30,31]. Moreover, in accordance with Stieger et al, our study shows that women are more inclined than men to undergo body art [31].

Fortunately, unlike what is reported in the scientific literature by Houghton et al [32], our investigation shows that only a limited number of freshmen have carried out body art on themselves and with improvised instruments. As pointed out by other authors, in most cases they turn to external operators [11,25,26,28].

The percentage of adolescents undergoing body decoration that reported complications might seem unalarming (13.2%); but when we consider that 9.2% of those treated at an authorized centre had complications, the percentage could be considered notable. Moreover, this finding fits well with that reported by Deschesnes et al [33] in a survey carried out in Quebec: while most teens say that a "professional" in a studio performed their body modification and that they received aftercare instruction, a high percentage of students reported health complications following the procedure.

The occupation of both mother and father does not affect the practice of body art. In fact, nowadays this practice has become customary, independent of social and cultural origin [26,34].

In terms of piercing, our data show that, in agreement with other works, the piercing is more common than tattooing [11,31,35]. Women are more inclined than men to have a piercing; in addition, females showed a higher interest than males in acquiring further piercings in the future. This fits well with work reported by Mayers et al [30] who examined a sample of university undergraduates in Pleasantville (NY, US). Our data concerning those who have stated to have never had a piercing, but were likely to consider it in the future are comparable to those of another group - Cegolon et al [34] - who examined a larger sample of young adolescents (4.277 students attending secondary schools).

The age at the first piercing is lower than the one of the first tattoo. A hypothesis for this difference could be given by the replies to the question on how to remove the piercing and the tattoo: procedures for removing a tattoo are more invasive than those for removing the piercing. In fact, 92.1% of the sample states that to remove the piercing it is sufficient to close the hole; 59.8% knew that a surgical procedure is necessary to remove the tattoo.

Although those who underwent piercing when they were underage are more than those who underwent tattooing when they were underage, the attitude towards informing the parents is quite similar.

The head is the favorite part of the body to place the piercing, while the limb is the favorite part of the body to place the tattoo. This finding seems to correspond with Antoszewski et al [11] examining body art location.

Those who live in urban areas are more inclined than those who live in smaller municipalities to undergo piercing; this finding is confirmed also by Antoszewski et al [29] in a survey among 968 Polish people living in Lodz.

In terms of tattoos, in contrast to piercing, women are not more inclined than men to have a tattoo and neither does the female show a higher interest than the male in acquiring tattoos in the future. This agrees with work reported by Mayers et al [30]. Nevertheless, our survey is comparable with data published by Stieger et al. [31], which indicated that many non-tattooed young adults would consider being tattooed in the future.

It is worth noting that, in contrast to Antoszewski et al [29], our survey shows that those who live in urban areas are more inclined than those who live in smaller municipalities to undergo tattooing. In general, comparing the tattoo group to the piercing group we can state that our survey shows that young adults coming from main towns are more likely to undergo body art.

In accordance with Stieger at al. [31], the average number of tattoos per tattooed participant seems lower than the average number of piercings per pierced participant.

The average age at the first tattoo was significantly higher than the average age at the first piercing and this result fits well with other work [26,29]; anyway, in our study, this result is affected by a large effect size.

One of the limits of the present study was that the questionnaire did not include a question for those who answered "I asked someone's advice" to specify by whom the advice was given. In fact, knowing who the advisor is could be important information to better target eventual educational interventions. Moreover, we should point out that the present study did not consider some variables that may influence the attitude to body art, such as political orientation or religious persuasion. As reported in the literature by Antoszewski et al [17], body ornamentation was connected to religious rituals and was a sign of social status. In any case, Stieger et al [31], in a previous study, demonstrated that there isn't any association between these variables and both piercing and tattoo practices.

## Conclusion

Although limited to freshmen, the present study confirms that the tendency to modify one's own body is spreading more and more among young people. Piercing and tattoos have now overcome most prejudices and the rather tawdry image that once accompanied them; in fact, nowadays the practice has become widespread, the social and cultural extraction not relevant [26,34]. Nevertheless, the sample investigated showed that there is still much lack of information on risks of this practice. For this reason, it is even more important to make information in this respect clearer, especially before the subject decides to undertake it [36].

Information about the freshmen's knowledge, attitudes and practices could help in effective planning for health promotion strategies; appropriate preventive measures should be adopted by professionals such as teachers, nurses, physicians and others who are in contact with young adults to help them make informed choices. Furthermore, it could be interesting to develop collaborative educational programs between body artists and schools, sharing information about body art in general, including the inherent risks, and encouraging young adults to contemplate their decisions carefully in advance.

## Competing interests

The authors declare that they have no competing interests.

## Authors' contributions

AQ, CN and MTM contributed equally to the definition of the study protocol, to the data collection, input and analysis and to the manuscript drafting and writing; GC contributed to the definition of the study protocol and to the data collection; CM contributed to the data collection and input; FF contributed to the data analysis. All authors read and approved the final manuscript.

## Pre-publication history

The pre-publication history for this paper can be accessed here:

http://www.biomedcentral.com/1471-2458/11/774/prepub
